# Impact of online group studying for the MRCPsych A exam amongst international doctors logging-in from 7 countries

**DOI:** 10.1192/bjo.2021.706

**Published:** 2021-06-18

**Authors:** Praveen Kumar, Sara Mohsen, Oksana Zinchenko, Philip Verde, Kathleen Breslin, Alice Judge, Sridevi Shanmugam

**Affiliations:** 1 NHS Highland; 2 Essex Partneship Universiy NHS Foundation Tust

## Abstract

**Aims:**

Recently, global-remote group studying has been made possible via digital video conferencing platforms. In preparation for the December 2020 MRCPsych part A exam, a study group was formed comprising 30 International Medical Gaduates (IMG) logging-in from different countries via 3 hour Zoom-study sessions hosted daily from 28th September until 12th December 2020 (1800-2100 GMT time). This study demonstrates the impact of online group study in preparation for the MRCPsych A exam for s via data collected through questionnaires.

**Method:**

The data of the study were collected through the questionnaires given to the group study members containing a total of 17 questions, 5 of which were open-ended.

The participants totalled 30 International Doctors who responded to an advertisement to form an online study group on Facebook. They logged-in for the sessions from seven different countries: Malaysia, India, Bangladesh, Ireland, Nigeria, Saudi Arabia, and the United Kingdom. The participants represented different working grades incuding experiences in psychiatry ranging from 0 to 5 years.

Data were analysed using percentage. The answers given to the open-ended questions were each examined using descriptive interpretation methods.

**Result:**

Thematic analysis demonstrated that online group study made learning faster and easier. 96.6% support using online study sessions for future exams citing that they fostered cooperation, respect for diverse opinions and motivation for regular studying. 93.1% and partly 6.9% found the experience enjoyable and enabled the cultivation of different ideas. Indeed, 89.7% relied on it as a big part of their preparation with 26 saying it contributed to their passing of the exam success.

Almost three quarter of participants in the group also forged friendships and a sense of trust. It also became a platform for expressing opinions comfortably and developing communication and interpersonal skills.

Different working hours and time zones represented a challenge with most linking in at odd hours. Cultural differences were ultimately accepted including aspects of delivery of information which made a few participants appear abrupt.

**Conclusion:**

With the ease in which social media connects us on a global scale, online study groups connecting IMGs from various backgrounds and diverse cultures not only makes exam preparations stimulating and easier to pass but also fosters interpersonal skills and connections that would be an asset in the long run.

